# Virucidal and Synergistic Activity of Polyphenol-Rich Extracts of Seaweeds against Measles Virus

**DOI:** 10.3390/v10090465

**Published:** 2018-08-30

**Authors:** Karla Morán-Santibañez, Mario A. Peña-Hernández, Lucia Elizabeth Cruz-Suárez, Denis Ricque-Marie, Rachid Skouta, Abimael H. Vasquez, Cristina Rodríguez-Padilla, Laura M. Trejo-Avila

**Affiliations:** 1 Laboratorio de Inmunología y Virología, Facultad de Ciencias Biológicas, Universidad Autónoma de Nuevo León, Ciudad Universitaria, CP 66455 San Nicolás de los Garza, NL, Mexico; mario.pena-hernandez@yale.edu (M.A.P.-H.); crrodrig07@gmail.com (C.R.-P.); 2 Department of Chemistry and Biochemistry, Border Biomedical Research Center, The University of Texas at El Paso, El Paso, TX 79968, USA; ahvasquez@miners.utep.edu; 3 Programa Maricultura, Facultad de Ciencias Biológicas, Universidad Autónoma de Nuevo León, Ciudad Universitaria, CP 66455 San Nicolás de los Garza, NL, Mexico; lucia.cruzsr@uanl.edu.mx (L.E.C.-S.); denisricque@hotmail.com (D.R.-M.); 4 Department of Biology, University of Massachusetts, Amherst, MA 01003, USA; rskouta@umass.edu

**Keywords:** virucidal, Polyphenol-rich extracts, Measles

## Abstract

Although preventable by vaccination, Measles still causes thousands of deaths among young children worldwide. The discovery of new antivirals is a good approach to control new outbreaks that cause such death. In this study, we tested the antiviral activity against Measles virus (MeV) of Polyphenol-rich extracts (PPs) coming from five seaweeds collected and cultivated in Mexico. An MTT assay was performed to determine cytotoxicity effect, and antiviral activity was measured by syncytia reduction assay and confirmed by qPCR. PPs from *Ecklonia arborea* (formerly *Eisenia arborea*, Phaeophyceae) and *Solieria filiformis* (Rhodophyta) showed the highest Selectivity Index (SI), >3750 and >576.9 respectively. Both PPs extracts were selected to the subsequent experiments owing to their high efficacy and low cytotoxicity compared with ribavirin (SI of 11.57). The combinational effect of PPs with sulphated polysaccharides (SPs) and ribavirin were calculated by using Compusyn software. Synergistic activity was observed by combining both PPs with low concentrations of *Solieria filiformis* SPs (0.01 µg/mL). The antiviral activity of the best combinations was confirmed by qPCR. Virucidal assay, time of addition, and viral penetration evaluations suggested that PPs act mainly by inactivating the viral particle. To our knowledge, this is the first report of the virucidal effect of Polyphenol-rich extracts of seaweeds.

## 1. Introduction

The discovery of antivirals with high efficacy, low cost, and low cytotoxicity is a long-pursued goal in drug discovery. The marine environment is a wealthy source of biological and chemical diversity of natural antivirals; the investigation of natural antivirals isolated from marine sources is an interesting approach in the development of new antiviral agents [1].

Screening assays of the antiviral activity of extracts from seaweeds have demonstrated the antiviral potency of chemical compounds present in these organisms [2]. Polyphenols are produced by most plants, including seaweeds, where they act as antioxidants that protect from external conditions such as stress and herbivores [3]. Some of the compounds found in seaweeds with the best antiviral activity are sulphated polysaccharides and phenolic compounds [4]. The antiviral activity of polyphenols has been observed mainly against enveloped viruses such as Retrovirus, Influenza Virus, Papilloma Virus, Herpes virus, and flaviviruses [5,6,7,8,9]. The Measles virus (MeV) is an enveloped virus that has regained importance as a pathogen as a result of the several recent outbreaks that have occurred in developed countries [10]. Despite the availability of a vaccine, this illness has not been eradicated; hence, the use of an effective antiviral as a therapy could contribute to outbreak containment and lead to eradication.

The strategy of simultaneously attacking multiple targets is a studied approach in the control of viral diseases [11]. Combining targeted therapies have demonstrated multiples advantages in this field, such the reduction of individual drug doses, the decrease in the side effects of antiviral agents, and the prevention of the emergence of drug-resistant viruses [12].

In the present study, we tested the antiviral activity in vitro against MeV of Polyphenol-rich extracts isolated from five Mexican seaweeds. We tested the combined antiviral effect of the best polyphenols with ribavirin and with sulphated polysaccharides isolated from the same seaweeds with potent antiviral properties [13]. The main goal of this research was to discover new candidates of antiviral drugs with a low cytotoxicity and affordable cost of production that could help control viral infection diseases.

## 2. Materials and Methods

### 2.1. Antiviral Agents

#### 2.1.1. Collection of Seaweeds

Five species of Mexican macroalgae were used in this study, three from Baja California (*Macrocystis pyrifera*, *Ecklonia arborea* (formerly *Eisenia arborea*), and *Silvetia compressa* (formerly *Pelvetia compressa*), one green seaweed from Southern Baja California (*Ulva intestinalis*), and one red seaweed from Yucatan (*Solieria filiformis*). In a previous study, our group reported in detail the collection of these five seaweeds [13].

*Macrocystis pyrifera* (Linnaeus) C. Agardh was collected in Bahía de Ensenada (Manto Jantay) in front of the Salsipuedes beach (31.983, −116.815), in January 2013. *Ecklonia arborea* J. E. Areschoug and *Silvetia compressa* (J. Agardh) De Toni were collected in the Escalera Zone, North of Punta China (31.520, −116.650) in December 2014–January 2015. The green alga *Ulva intestinalis* (Linnaeus) was collected from the water drainage channel of the Gran Mar shrimp farm, on the Baja California West coast (24.434, −111.584) in August 2014. *Solieria filiformis* (Kützing) P. W. Gabrielson, was obtained from an aquaculture facility at the Telchac Marine station-CINVESTAV, Yucatan (Mexico), where it is periodically cultivated in bimonthly cycles in semiopen tanks as part of an Integrated Multitrophic aquaculture system. The sample used came from a batch cultured from April to May 2014.

#### 2.1.2. Polyphenol-Rich Extracts Isolation

Polyphenol extraction was performed according to Xi et al. [14] with few modifications. Briefly, 10 g of alga powder was washed with distillated water and dried at room temperature overnight. The washed powder was extracted with 200 mL 50% *v*/*v* ethanol and sonicated (Ultrasonic cleaner 50HT, VWR International, West Chester, PA, USA) for 30 min at room temperature, followed with an extraction period in a bath shaker (Shak-R-bath, Lab-line, Melrose Park, IL, USA) at 70 °C during 2 h. The samples were centrifuged (IEC Centra MP4R, International equipment company, Needham, MA, USA) for 15 min (2500 rpm). The supernatant was recovered and added with 96% ethanol for residual polysaccharides precipitation, before centrifuging for 15 min (2500 rpm). The ethanol of the supernatant was evaporated at 55° in a rotary evaporator and the water was eliminated by freeze drying. Dried samples were suspended in Dulbecco’s modified Eagle’s medium (DMEM) (Gibco Invitrogen, Carlsbad, CA, USA) at a concentration of 2.5 mg/mL and filtered through a membrane filter (pore size, 0.45 mm).

### 2.2. Cell Line and Virus

Vero cells (green African monkey kidney cells) were purchased from the American Type Culture Collection (ATCC^®^ CCL-81™) (Manassas, VA, USA) and were grown at 37 °C in a 5% CO_2_ atmosphere in DMEM/F12 (DMEM/F12, Gibco Invitrogen, Carlsbad, CA, USA) supplemented with 5% (*v*/*v*) fetal bovine serum FBS, Gibco Invitrogen, Carlsbad, CA, USA) and 1% (*v*/*v*) antibiotic (Gibco Invitrogen, Carlsbad, CA, USA). Measles virus (Edmonston strain) was purchased from ATCC (ATCC^®^ VR-24TM). Virus was propagated on Vero cells and tittered by fifty percent tissue culture infectious dose (TCID_50_) assay, according to their cytopathic effect (CPE). Aliquots of viral stock were stored at −80 °C until use.

### 2.3. Cytotoxicity Assay

The cytotoxicity of the compounds was evaluated by MTT reduction assays. The cells were cultured in 96-well plates at a density of 1.5 × 10^4^ cells/well at 37 °C in and 5% CO_2_. Increasing concentrations of PPs diluted in DMEM (Gibco Invitrogen, Carlsbad, CA, USA) were added; after 48 h of incubation the media were replaced with MTT solution. After 1 h 30 min 150 µL of DMSO was added to dissolve the formazan crystals and incubated at room temperature for 15 min. The optical density (OD_450_ nm) was measured using a microplate reader (Multiskan FC, Thermo, Waltham, MA, USA). The cytotoxicity was expressed by percentage as the mean value of three independent experiments considering control cells absorbance as 100% viable. CC_50_ was the concentration of the test substances that inhibited the Vero cells growth by 50% compared with the growth of the untreated cells. All variables were performed in triplicate and repeated at least three times (N = 9).

### 2.4. Antiviral Activity

The antiviral activity of PPs against MeV was evaluated by syncytia reduction assays. Vero cells seeded in 12-well plates were treated with different concentrations of PPs (0.01–5 µg/mL), and infected with MeV (1 × 10^3.5^ TCID_50_ of Edmonston strain) at the same time for 1 h at 37 °C, allowing the virus to adsorb. After the incubation period, Vero cells were washed with PBS. The assays were performed by adding the compounds during all the infection cycle, i.e., after PBS washing; the compounds were added again and remained until the end of the experiment. Each treatment was tested in triplicate, and each experiment was performed at least in triplicate. After incubation period (48 or 72 h at 37 °C in a 5% CO_2_) monolayers were fixed with methanol:acetone and stained with 1% crystal violet in order to observe CPE and count syncyta. The result was expressed as a percentage of total syncytia observed in viral control monolayers (untreated cultures). The IC_50_ was determined from dose-response curves and the selectivity index (SI) values were calculated as CC_50_/IC_50_. PPs with the best SI were selected for the following experiments. All variables were performed in triplicate and repeated at least three times (N = 9).

### 2.5. Quantitative Real-Time PCR

Total RNA was isolated using RNAzol^®^ RT (MRC Inc., Cincinnati, OH, USA) from treated (1 and 5 µg/mL) and untreated Vero cells. Reverse transcription was performed using the High Capacity cDNA Reverse Transcription Kit (Applied Biosystems, Foster City, CA, USA) and the viral genome was amplified with specific primers (MeVF: 5′ GAGGGTCAAACAGAGTCGAG 3′, MeVR: 5′ CGGTTGGAAGATGGGCAG 3′). The real-time PCR was carried out using SensiFASTTM SYBR^®^ No-ROX Kit (BIOLINE, Memphis, TN, USA) and the StepOnePlus Real-Time PCR Systems (Applied Biosystems, Foster City, CA, USA) with the following procedures: 95 °C for 2 min, followed by 40 cycles of 95 °C for 2 s, 60 °C for 10 s, and 72 °C for 20 s. The number of viral copies was calculated using a standard curve (reference) and results were expressed as a percentage of total number of viral copies observed in viral control monolayers (untreated cultures). All variables were performed in triplicate and repeated at least three times (N = 9).

### 2.6. Evaluation of PPs Synergy

The combined effect on MeV infection of selected PPs with SPs (with antiviral activity and extracted from the same seaweeds) and ribavirin was evaluated. Each combination was tested on its corresponding IC_25_ and IC_50_ using syncytia reduction assays. The synergistic effect of combinations was calculated by using the CompuSyn software which calculates a combination index (CI) described by Chou [15]. As Chou indicated, CI values lower than 0.9 indicate synergism, CI values from 0.9 to 1.1 indicate an additive effect, and CI values higher than 1.1 indicate antagonism. Inhibitory activity of the combinations with the best synergistic effect was confirmed by qPCR assays. All variables were performed in triplicate for each experiment and repeated at least three times (N = 9).

### 2.7. Virucidal Assays

Virucidal activity of PPs against MeV was determined using syncytia reduction assays with monolayers of Vero cells grown in 12-well plates. The assays where assessed by adding the PPs (5 μg/mL) to an equal volume to MeV (1 × 10^3.5^ TCID_50_ of Edmonston strain) After 0, 1, 3 and 6 h of incubation, the mixtures were added to Vero cells and further incubated 1 h at 37 °C. After that time, the mixtures were removed and media was added. After incubation period (48 or 72 h at 37 °C in a 5% CO_2_) monolayers were fixed with methanol:acetone and stained with 1% crystal violet in order to count syncyta. The result was expressed as a percentage of total syncytia observed in viral control monolayers (untreated cultures). All variables were performed in triplicate for each experiment and repeated at least three times (N = 9).

### 2.8. Time of Addition Assays

Vero cell monolayers were infected with MeV (1 × 10^3.5^ TCID_50_ of Edmonston strain). PPs (5 µg/mL) were added at different times of infection: 60 min before infection and 0, 15, 30, 60, and 120 min after infection. Vero cells were incubated with each treatment for 1 h and then washed three times with PBS. After incubation period of 48 h at 37 °C and 5% CO_2_ monolayers were fixed with methanol:acetone and stained with 1% crystal violet; syncytia were counted subsequently and the result was expressed as a percentage of total syncytia observed in viral control monolayers. All variables were performed in triplicate for each experiment and repeated at least three times (N = 9).

### 2.9. Viral Penetration Assays

MeV penetration into Vero cells was evaluated according to the method reported by Huang and Wagner [16]. Vero cells grown in 12-well plates were precooled at 4 °C for 3 h and were infected with MeV (1 × 10^3.5^ TCID_50_ of Edmonston strain) at 4 °C for 1 h of incubation in the absence of PPs. Thereafter monolayers were washed three times with ice-cold PBS. Different concentrations of PPs (1 and 5 µg/mL) were added and the temperature was shifted to 37 °C, then incubated per 1 h at this temperature. Afterwards, incubation period cells were treated with 40 mM citrate buffer (pH 3.0) to inactivate non-penetrated viruses. Thereafter, buffer was replaced by DMEM and monolayers were incubated for 48 h at 37 °C and 5%CO_2_ and stained with 1% crystal violet; syncytia were counted subsequently. All variables were performed in triplicate for each experiment, and repeated at least three times (N = 9).

### 2.10. Total Phenolic Content (TPC) Evaluation

The total phenolic content (TPC) was measured using the Folin-Ciocalteu method, which utilizes Gallic acid (GA) as a standard reagent [17]. The Polyphenol-rich extracts were prepared in methanol, with a final concentration of 10 mg/mL. GA stock solution was prepared in methanol to provide the standard reference curve according to Skouta et al. [18]. Briefly, 100 µL each seaweed extract was transferred into a 1.5 mL Eppendorf tube and mixed with 200 µL of Folin-Ciocalteu reagent (10%), homogenized for 15 s (Standard Heavy-Duty Vortex Mixer VWR, Radnor, PA, USA), before adding 800 µL of sodium carbonate (700 mM in DI water). The tubes were allowed to stand for 2 h in complete darkness and 200 µL of each sample reaction was transferred to a 96-well microplate and absorbance was registered at 765 nm in a microplate reader (Epoch 2, BioTek Instruments Inc., Winooski, VT, USA). TPC were determined by comparison of the values obtained with the calibration curve of GA (R² = 0.999). The results were expressed as mg GA equivalents (GAE)/L.

### 2.11. High Performance Liquid Chromatography (HPLC) and Mass (MS) Analysis

The characterization of Polyphenol-rich extracts was carried out using high performance liquid chromatography (HPLC). Samples were suspended in LC-MS metanol and filtered through a membrane filter (pore size, 0.45 mm). The separation was achieved on a SunFire (Waters, Milford, MA, USA) C18 5 μm 4.6 × 150 mm column at ambient temperature using a Waters 2487 instrument (Waters, Milford, MA, USA). The mobile phase consisted of acetonitrile with 3% Acetic Acid (solvent A), water with 3% Acetic Acid (solvent B). The gradient used to separate *Solieria filiformis* extract was: 100% A at 0 min, 90% A and 10% B at 3.5 min, 50% A and 50% B at 5 min, and 100% B at 10–20 min. To separate *Ecklonia arborea* extract we used the following gradient: 100% A at 0 min, 50% A and 50% B at 2 min, 25% A and 75% B at 5 min, and 100% B at 7–20 min. The flow rate was 1.5 mL/min for 20 min at an injection volume of 10 μL. Once collected, the fractions were, dried and resuspended in methanol for mass analysis. Liquid chromatography/mass spectra (LC-MS) [+ESI] were taken on a JEOL AccuTOF TC-100 Mass Spectrometer (JEOL Ltd., Tokyo, Japan).

### 2.12. Statistical Analysis

Data were analyzed with SPSS 20 software. All variables were performed in triplicate for each experiment and repeated at least three times (N = 9). CC_50_ and IC_50_ values at 48 h were determined by Probit regression analysis. One-way ANOVA with Dunnet’s post hoc test was used for comparisons vs. viral control. Two-way ANOVA followed by a Tukey analysis were performed when comparing different techniques. The results of were considered significantly different if *p* < 0.05.

## 3. Results

### 3.1. Cytotoxicity and Antiviral Activity In Vitro of Polyphenol-Rich Extracts of Seaweeds (PPs)

**Figure 1:**
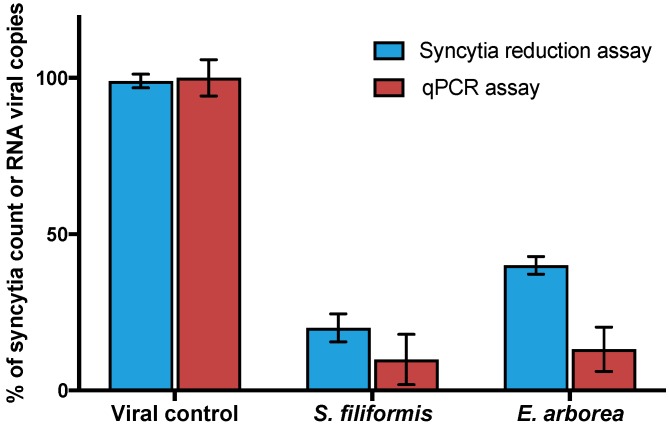
Antiviral activity of *Ecklonia arborea* and *Solieria filiformis* extracts. PPs were tested at 5 µg/mL by syncytia reduction and qPCR assays. Syncytia count and viral RNA copies number are given in % of the untreated control values. Each bar represents the average of three replicates.

To determine the cytotoxicity of PPs, an MTT assay was performed. Results indicated no relevant cytotoxicity for any of the PPs tested; CC_50_ could not be determined for most PPs because of the lack of cytotoxicity at tested concentrations (0.1 to 1500 µg/mL). On contrary ribavirin exhibited a CC_50_ of 405 µg/mL. Antiviral activity of PPs and ribavirin against MeV was evaluated by syncytia reduction assays at different concentrations (0.01, 0.1, 1, and 5 µg/mL of each PPs and 10, 20, 30, 40 and 50 µg/mL of ribavirin). As shown in Table 1 with calculated Selectivity Index values (SI), all tested compounds showed antiviral activity. PPs of *Ecklonia* (formerly *Eisenia*) and *Solieria* showed the best SI values and therefore were selected for the next experiments. As shown in Figure 1, antiviral activity of selected PPs was confirmed by qPCR assays and results were consistent with those observed by Syncytia reduction assays.

### 3.2. Combined Effect of Polyphenol-Rich Extracts (PPs) with Sulphated Polysaccharides (Sps) and Ribavirin

**Figure 2:**
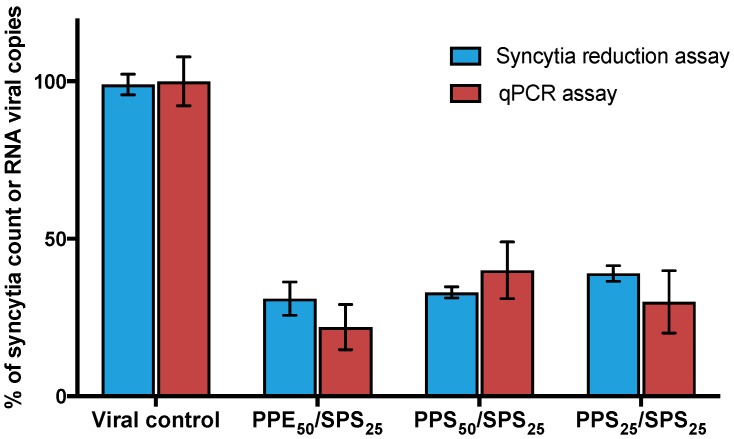
Antiviral activity of synergistic combinations of PPs and SPs. PPs and SPs were combined with their IC_25%_ and IC_50%_ and the antiviral effect was determined by syncytia reduction and qPCR assays. Syncytia count and viral RNA copies number are given in % of the untreated control values. Each bar represents the average of three replicates.

The antiviral effect of PPs in combination with SPs (with antiviral activity tested and extracted of the same seaweeds) [13] and ribavirin was assessed by syncytia-reduction assays. Inhibitory concentrations 50% and 25% (IC_50_ and IC_25_) of PPs were tested with their corresponding IC_50_ and IC_25_ of SPs and ribavirin. Combinational Index values of all combinations were calculated using the CompuSyn software [15]. Combined effect of PPs and SPs is given in Table 2, where 10 of the 16 combinations showed synergism. Combinations of PPs from *Ecklonia arborea* (formerly *Eisenia*) as well as PPs from *Solieria filiformis* with SPs from *Solieria filiformis* showed the best synergistic effects and were confirmed by qPCR (Figure 2). IC_50_ of *Ecklonia arborea* (formerly *Eisenia*) PPs and IC_25_ of *Solieria filiformis* SPS combination (PPE_50_/SPS_25_) showed the best synergistic effect. By contrast, Table 3 indicates the combined effect of PPs with ribavirin, as shown in the table; all the tested combinations were antagonic.

### 3.3. Virucidal Activity of Polyphenol-Rich Extracts (PPs)

**Figure 3:**
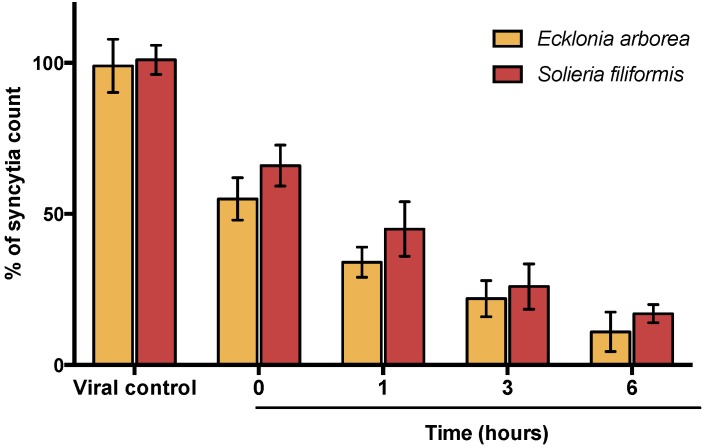
Virucidal effect of PPs. Vero cells were infected with MeV previously exposed to PPs at different times (0, 1, 3 and 6 h). Syncytia count is given in % of the untreated control values. Each bar represents the average of three replicates.

A virucidal assay was performed to analyze if the compounds act directly on the virus particle leading to infectivity inactivation. Virucidal activity was tested at 5 µg/mL in both PPs. Results determined that inhibitory activity increases directly proportional to the time of between the extracts and virus (Figure 3). Inhibitory activity observed at 6h leads to 83–89% of inhibition. Therefore, both PPs (*Ecklonia arborea* and *Solieria filiformis*) have a potential virucidal activity by inactivating viral particles.

### 3.4. Effect of Polyphenol-Rich Extracts (PPs) at Different Times of Addition

**Figure 4:**
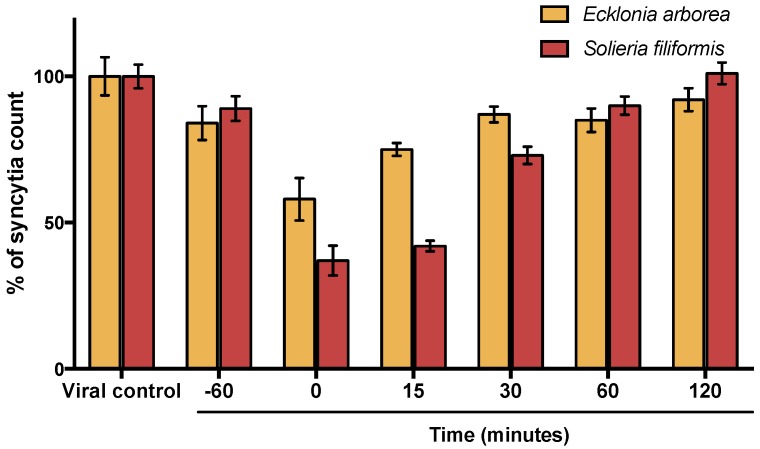
Time of addition assay. Antiviral activity of PPs was tested at different times of infection and analyzed by syncytia reduction assays. PPs were added to the cells 60 min before infection and at 0, 15, 30, 60, and 120 min after infection. Syncytia count is given in % of the untreated control values. Each bar represents the average of three replicates.

Time of addition experiments were performed to determine which step of the MeV cycle was targeted by PPs. Vero cells were infected with MeV and compounds were added at different times (60 min before infection and 0, 15, 30, 60, and 120 min after infection). As shown in Figure 4, results determined PPs from *Ecklonia arborea*, as well as PPS from *Solieria filiformis*, have their best antiviral activity at the first minutes of infection (0–15 min). Inhibitory activity observed at different times was lower than the observed in virucidal assays (6 h). The activity in the first minutes of infection suggests that inhibitory activity of PPs is possible due to a direct inactivation of the viral particle.

### 3.5. Effect of Polyphenol-Rich Extracts (PPs) in Viral Entry

**Figure 5:**
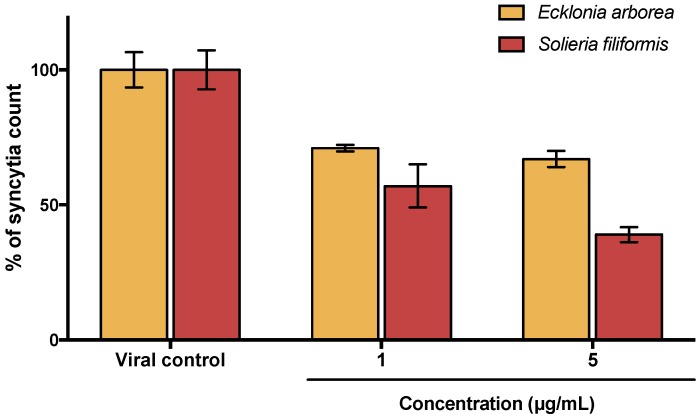
Effect of PPs on viral penetration. Vero cells were infected with MeV at 4 °C in the absence of PPs and then shifted to 37 °C to permit penetration of the adsorbed virus in the presence of PPs. Syncytia count is given in % of the untreated control values. Each bar represents the average of three replicates.

A viral entry assay was performed to determine whether entry events, downstream of virus binding, were inhibited by PPs. Monolayers were incubated with MeV at 4 °C for 1 h to allow virus binding but no viral entry. The unbound virus was inactivated with citrate buffer, and PPs (1 µg/mL or 5 µg/mL) were added to the cells and incubated at 37 °C in a 5% CO_2_. As shown in Figure 5, the best inhibitory effect was observed with *S. filiformis* PPs (5 µg/mL), compared with the results in untreated cells.

### 3.6. Estimated Total Phenolic Content (TPC)

The Folin–Ciocalteu method was used to determine total phenolic content of *Ecklonia arborea* and *Solieria filiformis* PPs. The TPC of each extract was determined using a regression equation of the calibration curve and expressed as gallic acid equivalents (GAE). Estimated TPC *of Ecklonia arborea* and *Solieria filiformis* were 179.16 ± 11.38 (GAE)/L and 102.22 ± 15.10 (GAE)/L respectively.

### 3.7. Polyphenol-Rich Extracts (PPs) Characterization by High Performance Liquid Chromatography (HPLC) and Mass (MS) Analysis

**Figure 6:**
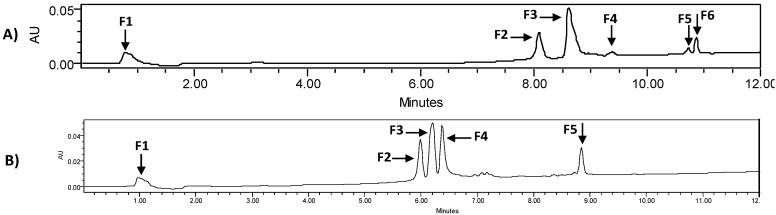
Chromatograms obtained for: (**A**) *Solieria filiformis* PPs; (**B**) *Ecklonia arborea* PPs, detection at 270 nm. The fractions collected are numbered (F1–F6) as indicated.

After optimizing HPLC conditions, we identified and collected six potential fractions in *Solieria filiformis* PPs (Figure 6A) and five fractions in *Ecklonia arborea* PPs (Figure 6B). Once collected, fractions were submitted to mass analysis. To identify tentative compounds in each isolated fraction based on mass spectra, we used the European MassBank, Phenol-Explores 3.6, and MassBank of North America databases [19,20,21]. The potential identification of each fraction from Polyphenol-rich extracts is shown in Table 4 and Table 5.

## 4. Discussion

The research of biological activities of marine products has yielded many bioactive compounds showing various pharmaceutical properties [23]. The demand for new antiviral agent discovery against emergent and re-emergent viruses has grown due to recent outbreaks. For this reason, the aim of the present study was to evaluate the antiviral activity of Polyphenol-rich extracts isolated from Mexican seaweeds against Measles virus.

One of the major challenges in the development of new antivirals is to find a compound with no cytotoxicity. Most of the Polyphenol-rich extracts tested in this study did not demonstrate cytotoxicity activity in Vero cells at high concentrations (Table 1). The lack of cytotoxicity of PPs isolated from seaweeds in Vero cells was also reported by Namvar et al., through testing of phlorotanins extracted from *Sargassum muticum* (Phaeophyceae) at concentrations lower than 200 µg/mL [24].

The use of secondary metabolites of algae as antiviral agents has been tested for a large number of enveloped viruses of medical and veterinary importance [25,26,27,28]. Seaweeds sulphated polysaccharides have also been extensively studied for antiviral activity, but seaweeds polyphenols are relatively new to antiviral research [29,30,31]. All of the PPs evaluated showed antiviral activity against MeV (Table 1). Polyphenol-rich extracts of *Solieria filiformis* and *Ecklonia arborea* (formerly *Eisenia arborea*) showed the highest Selectivity Index (>3750 and >576.9 respectively) and were selected to the subsequent experiments due to their high efficacy and low cytotoxicity by comparing with ribavirin, an FDA approved antiviral (SI of 11.57). Syncytia formation and viral titration by qPCR were used to evaluate antiviral activity of both extracts (Figure 1). A significant reduction between controls and treatments was observed with both techniques, but substantial significance differences were observed by comparing the highest concentrations tested of PPs in both techniques. Although PCR is typically a more sensitive method than tissue culture techniques, the presence of viral RNA may not always reflect an association with infective viruses production [32].

Synergistic activity of secondary metabolites of seaweeds has been reported for our group. We previously tested the combinational activity of sulfated polysaccharides (SPs) isolated from the same five seaweeds and observed a synergistic effect because of the different mode of action in the SPs evaluated [13]. In this study, we tested the antiviral activity of PPs in combination with SPs extracted of the same seaweeds and ribavirin. The combinations with the best synergistic activity were observed by combining PPs of *Solieria filiformis* as well as *Ecklonia arborea* PPs with SPs of *Solieria filiformis* at low concentrations (0.01 µg/mL) (Table 2). The antiviral activity of the best combinations was confirmed by qPCR (Figure 2). All of the evaluated combinations of PPs with ribavirin showed an antagonic effect (Tale 3). Owing to the synergistic effect observed by combining PPs and SPs, we questioned the different mode of action between both extracts; therefore, we assayed three different techniques to elucidate the mode of action of PPs. The virucidal activity of PPs of *Solieria filiformis* and *Ecklonia arborea* was tested in order to determine if the compounds are inactivating the virus before the infection of Vero cells (Figure 3). Both polyphenols were shown to have a remarkable inhibitory effect at minute 0 and 15 of the infection (Figure 4); a decrease in syncytia and viral load was found in the viral penetration tests (Figure 5); the best inhibitory effect was observed when performing the virucidal test, decreasing to 90% the formation of syncytia after 6 h of PPs-virus interaction (Figure 3). It is possible that the effect of these Polyphenol-rich extracts is the direct deactivation of the virion, which therefore, prevents it from adsorbing and penetrating the host cell, an effect that we consistently see in the addition and penetration times tests. Virucidal activity of polyphenols has been observed previously, such as, polyphenols isolated from *Cistus* (Tracheophyta, Magnoliopsida), a floral plant that showed virucidal effect against Influenza virus through inhibition of HA binding to cellular receptors [33].

The total phenolic content test confirmed that both extracts are rich in phenolic compounds. HPLC and mass analysis allowed us to identify potential natural compounds that may be playing an important role in the antiviral activity of the extract. Six fractions and five fractions were collected from *Solieria filiformis* and *Ecklonia arborea* extracts, respectively (Figure 6A,B). Tentative compounds identified in *Solieria filiformis* extract are mostly phenolic compounds previously reported as secondary metabolites of seaweeds (Table 4). Quercetin and kaempherol were reported in high percentages in the red seaweed *Gracilaria dendroides* (Rhodophyta), which showed antimicrobial activity [34]. High concentrations of quercetin were found in a polyphenol-rich extract isolated from the red seaweed *Kappaphycus alvarezii* (formerly *Eucheuma cottonii*) which suppressed breast tumor via hormone modulation and apoptosis induction [35]. Antiviral activity of quercetin and kaempherol has been reported against Herpes Simplex Virus and Influenza virus, as well as the synergistic antiviral effect [36,37]. Phlorofucofuroeckol-B, a compound identified in the *Ecklonia arborea* (formerly *Eisenia arborea*) extract, is a phlorotannin previously reported in this brown seaweed (Table 5). Phlorofucofuroeckol-B, isolated from *Ecklonia bicyclis* (formerly *Eisenia bicilys*) and *Ecklonia arborea*, has also been reported as showing potent antioxidant and anti-allergic [38,39]. Antiviral activity in vitro of phlorofucofuroeckol-A isolated from *Ecklonia bicilys* (formerly *Eisenia bicilys*) against murine norovirus was reported to have a Selectivity index of 668.87 [31]. Even though there are some reports about the antiviral activity of seaweeds polyphenols that prevent viral adsorption and replication [40], this is the first report of the virucidal effect of Polyphenol-rich extracts of seaweeds, to our knowledge.

## 5. Conclusions

In summary, our study demonstrates that Polyphenol-rich extracts isolated from Mexican seaweeds have significant virucidal activities against Measles virus in vitro. Virucidal activity of the extract is not only a prophylactic strategy before viral infection, but can also be successful as a treatment after infection, avoiding virus dissemination. The synergistic effect shown with sulphated polysaccharides proposed a desirable therapeutic effect, reducing the concentration of the compounds, thereby also their cellular toxicity, and avoiding the resistance of the virus to the action of these compounds.

## Figures and Tables

**Table 1:** Cytotoxic effect, antiviral activity and selectivity index of PPs.

Extract or Compound ^a^	CC_50_ (μg/mL) ^b^	IC_50_ (μg/mL) ^c^	SI ^d^
*Macrocystis pyrifera* (Phaeophyceae) PP	>1500	3 ± 0.33	>500
*Ecklonia arborea* (formerly *Eisenia arborea*, Phaeophyceae) PP	>1500	2.6 ± 0.28	>576.9
*Silvetia compressa* (formerly *Pelvetia compressa*, Phaeophyceae) PP	165.03 ± 9.7	1.86 ± 0.61	306.9
*Ulva intestinalis* (Chlorophyta) PP	>1500	3.1 ± 0.23	>483.9
*Solieria filiformis* (Rhodophyta) PP	>1500	0.4 ± 0.11	>3750
Ribavirin	405 ± 4.1	35 ± 1.8	11.57

**^a^** Polyphenol-rich extracts of seaweeds or compound. **^b^** Concentration of test compound (μg/mL) that reduced Vero cell viability by 50%. **^c^** Concentration of a test compound that reduced the number of MeV syncytia in Vero cells by 50%. **^d^** Selectivity index value.

**Table 2:** Combined antiviral effect of PPs and SPs of *Ecklonia arborea* and *Solieria filiformis.*

Combination	Compound Concentration (μg/mL)				% Relative Syncytia of Each Combination	SD	CI	Description
	Polyphenol PPE or PPS *		Polysaccharides SPE or SPS *					
	*Ecklonia arborea*	*Solieria filiformis*	*Ecklonia arborea*	*Solieria filiformis*				
PPE_50_-SPE_50_	2.6	-	0.275	-	38.8	3.5	0.79	Synergism
PPE_25_-SPE_50_	0.03	-	0.275	-	49.1	6.8	1.75	Antagonism
PPS_50_-SPE_50_	-	0.4	0.275	-	55.1	8.1	4.4	Antagonism
PPS_25_-SPE_50_	-	0.07	0.275	-	36.7	2.9	0.65	Synergism
PPE_50_/SPE_25_	2.6	-	0.01	-	40	6.4	0.19	Synergism
PPE_25_/SPE_25_	0.03	-	0.01	-	73.5	2.6	3.6	Antagonism
PPS_50_/SPE_25_	-	0.4	0.01	-	44.9	3.3	0.4	Synergism
PPS_25_/SPE_25_	-	0.07	0.01	-	57.14	6.7	0.4	Synergism
PPE_50_/SPS_50_	2.6	-	-	0.985	22.5	6.2	0.57	Synergism
PPE_25_/SPS_50_	0.03	-	-	0.985	34.5	8.3	1.85	Antagonism
PPS_50_/SPS_50_	-	0.4	-	0.985	28.8	1.5	1.37	Antagonism
PPS_25/_SPS_50_	-	0.07	-	0.985	36.7	2	2.25	Antagonism
PPE_50_/SPS_25_	2.6	-	-	0.01	30.6	5.3	0.03	Synergism
PPE_25_/SPS_25_	0.03	-	-	0.01	42.9	1.1	0.4	Synergism
PPS_50_/SPS_25_	-	0.4	-	0.01	33.3	1.8	0.03	Synergism
PPS_25_/SPS_25_	-	0.07	-	0.01	38.8	2.5	0.11	Synergism

* PPE and PPS correspond to polyphenol-rich extract of *Ecklonia arborea* and *Solieria filiformis* respectively; * SPE and SPS correspond to polysaccharides of *Ecklonia arborea* and *Solieria filiformis* respectively.

**Table 3:** Combined antiviral effect of PPs of *Ecklonia arborea* and *Solieria filiformis* with ribavirin.

Combination	Concentration (μg/mL)			% Relative Syncytia of Each Combination	SD	CI	Description
	Polyphenols PPE or PPS *		Ribavirin				
	*Ecklonia arborea*	*Solieria filiformis*					
PPE_50_/R_50_	2.6	-	35	4	0.5	9.1	Antagonism
PPE_50_/R_25_	2.6	-	16	32	1.1	2.6	Antagonism
PPE_25_/R_50_	0.03	-	35	20	1.8	1.3	Antagonism
PPE_25_/R_25_	0.03	-	16	33.2	1.5	1.2	Antagonism
PPS_50_/R_50_	-	0.4	35	13.6	1.2	3.6	Antagonism
PPS_50_/R_25_	-	0.4	16	36.4	1.9	2.5	Antagonism
PPS_25_/R_50_	-	0.07	35	24.3	2.1	2.2	Antagonism
PPS_25_/R_25_	-	0.07	16	30.3	1.8	1.3	Antagonism

* PPE and PPS correspond to polyphenol-rich extract of *Ecklonia arborea* and *Solieria filiformis* respectively.

**Table 4:** HPLC Fractions of Polyphenol-rich extract isolated from *Solieria filiformis*.

Fraction	Retention Time (min)	Major Fragment Ions *m*/*z* (% Base Peak)	Tentative Identification
F1	0.802	298.339 (100), 136.989 (92.09), 150.115 (91.36)	5-Methylthioadenosine ^a,b^ Inosine ^a,b^, L-Methionine ^a^
F2	8.109	230.243 (100)	Terbutylazine ^a,b^
F3	8.620	507.299 (100), 683.430 (98.13), 639.405 (97.21), 551.349 (95.74), 595.376 (91.57)	Quercetin 3-(6-O-acetyl-beta-glucoside) ^a,b^, Methyllycaconitine ^a^^,^^b^, Demethoxycentaureidin 7-O-rutinoside^ab^, Quercetin 3-O-(6′′-malonyl-glucoside) ^a,b,c^, Kaempferol-3-O-rutinoside ^a,b,c^.
F4	9.382	301.134 (100)	Kaempferide^a^
F5	10.716	413. 256 (100)	7-acetyloxy-2-(3,4-diacetyloxyphenyl)-4-oxochromen-5-yl acetate ^a^
F6	10.860	264.237 (100)	Abscisic acid ^a,b^

^a^ Confirmed with Mass Spectroscopy (MS) fragmentation and European MassBank results; ^b^ Confirmed with Mass Spectroscopy (MS) fragmentation and MassBank of North America results; ^c^ Confirmed with Mass Spectroscopy (MS) fragmentation and Phenol-explorer of North America results.

**Table 5:** HPLC Fractions of Polyphenol-rich extract isolated from *Ecklonia arborea*.

Fraction	Retention Time (min)	Major Fragment Ions *m*/*z* (% Base Peak)	Tentative Identification
F1	1.052	365. 125(100), 205.064 (98.23), 601.138 (40)	Cellobiose ^a,b^, Tryptophan ^a^ Phlorofucofuroeckol-B ^c^
F2	5.982	602.140 (100), 268.993 (73.23), 230.244 (68.75)	Phlorofucofuroeckol-B ^c^, Formononetin ^a,b^, Apigenin 7-O-glucoside ^a^
F3	6.195	413.259 (100), 327.195 (67.57)	Leganin ^a^ Feruloyl tartaric acid ^a^
F4	6.733	205.074 (100)	Tryptophan ^a^
F5	8.844	601.139 (100)	Phlorofucofuroeckol-B ^c^

^a^ Confirmed with Mass Spectroscopy (MS) fragmentation and European MassBank results; ^b^ Confirmed with Mass Spectroscopy (MS) fragmentation and Phenol-Explores 3.6 results; ^c^ Confirmed based on Choi et al. [22].
